# Regulation of microglial activation by progranulin through the NF-κB pathway in subarachnoid hemorrhage and its effect on white matter injury

**DOI:** 10.1016/j.neurot.2026.e00924

**Published:** 2026-05-20

**Authors:** Ziliang Hu, Pan Lv, Wenting Lan, Menglu Shen, Yi Huang, Chengzhi Chen, Yan Zheng, Kaixuan Chen, Xiang Gao, Wei Cui, Chenhui Zhou

**Affiliations:** a Department of Neurosurgery, Ningbo Key Laboratory of Nervous System and Brain Function, The First Affiliated Hospital of Ningbo University, Ningbo, Zhejiang 315010, China; b Department of Clinical Laboratory, The First Affiliated Hospital of Ningbo University, Ningbo, Zhejiang 315010, China; c Department of Radiology, Department of Neurosurgery, Ningbo Key Laboratory of Nervous System and Brain Function, The First Affiliated Hospital of Ningbo University, Ningbo, Zhejiang 315010, China; d Cixi Third People's Hospital, Cixi, Zhejiang 315324, China; e Department of Neurosurgery, Ningbo Hangzhou Bay Hospital, Ningbo, Zhejiang, China; f Department of Neurosurgery, Ningbo Branch, Ren Ji Hospital, Shanghai Jiao Tong University School of Medicine, Ningbo, Zhejiang 315336, China

**Keywords:** SAH, PGRN, Myelin, Microglia

## Abstract

Subarachnoid hemorrhage (SAH) is a severe cerebrovascular condition commonly associated with white matter injury and subsequent cognitive dysfunction. Emerging evidence suggests that progranulin (PGRN) is involved in neuroinflammation regulation and myelin integrity maintenance. This study aimed to investigate the role of PGRN in SAH and to elucidate its potential neuroprotective mechanisms. Magnetic resonance imaging (MRI) and cerebrospinal fluid (CSF) samples from clinical patients were analyzed, with inflammatory factors measured by enzyme-linked immunosorbent assay (ELISA). In a rat SAH model, myelin basic protein (MBP) and ionized calcium-binding adapter molecule 1 (IBA-1) expression were assessed by Western blotting and immunofluorescence, and transcriptomic analysis was performed to identify gene expression changes. Cognitive function was evaluated using the Morris water maze. Transcriptomic results showed that genes altered after SAH were mainly enriched in pathways related to glial cell differentiation, myelin integrity, and immune regulation. Clinical MRI and CSF findings suggested an association between SAH, inflammation, and cognitive impairment. In animal experiments, increased PGRN expression was associated with reduced brain injury and improved myelin integrity. Behavioral testing indicated that treatment with the PGRN enhancer AL001 shortened escape latency and increased time spent in the target quadrant, suggesting improved cognitive performance. These findings suggest that PGRN may exert neuroprotective effects after SAH by modulating glial activity and maintaining myelin integrity, and that AL001 may represent a potential therapeutic approach for alleviating cognitive dysfunction following SAH.

## Graphical abstract

**Figure:**
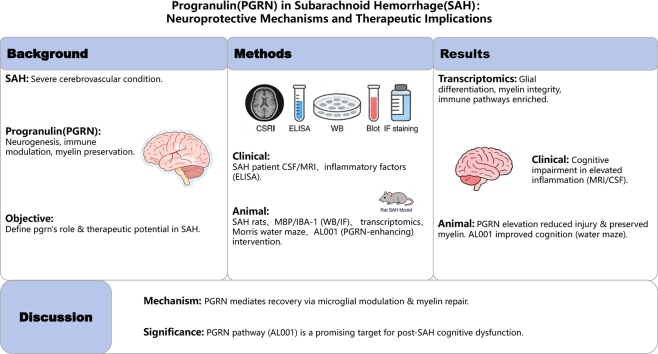

## Introduction

Subarachnoid hemorrhage (SAH) represents a severe and often life-threatening subtype of hemorrhagic stroke, characterized by bleeding into the subarachnoid space, which can lead to sudden neurological deterioration and significant long-term complications, responsible for around 5–10% of all stroke occurrences. It is associated with a notably high rate of mortality and long-term disability. Typically, SAH presents as an acute event, and it is often followed by an early phase of brain injury (EBI). This initial brain injury is a critical factor contributing to the poor prognosis of individuals affected by SAH, as it sets the stage for further complications such as inflammation, oxidative stress, and secondary injury, all of which severely impact the recovery process, a major factor contributing to the high mortality and disability rates in SAH patients. Research has demonstrated that early brain injury (EBI) is a complex pathological condition involving the interaction of multiple potential mechanisms, including but not limited to neuroinflammatory responses, oxidative stress, and ferroptosis [[1], [2], [3]]. The activation of microglia and the resulting neuroinflammatory response are considered to be crucial drivers of EBI(4). Within the first 72 h after SAH, activated microglia secrete large quantities of pro-inflammatory cytokines, amplifying the inflammatory cascade and contributing to neuronal damage. Current research has found that microglia can exhibit different functions under various pathophysiological conditions, a phenomenon referred to as disease-associated microglia [4]. Therefore, this study aims to investigate the changes and roles of different microglial activation states during the course of SAH, and to explore whether regulating microglial activation after SAH can alleviate white matter myelin damage.

Brain white matter primarily consists of axons ensheathed in myelin, nerve fiber bundles, and glial cells, and is responsible for neural signal transmission between different brain regions as well as connecting the central and peripheral nervous systems. The structural integrity of myelin is crucial for the proper function of white matter. Damage to myelin is a primary manifestation of white matter injury and can lead to cognitive dysfunction, motor deficits, and sensory disturbances, among other neurological impairments [5]. Myelin development and maintenance are essential for normal nervous system function, playing a vital role in neural development and spanning the entire physiological process of the nervous system.

**Fig. 1:**
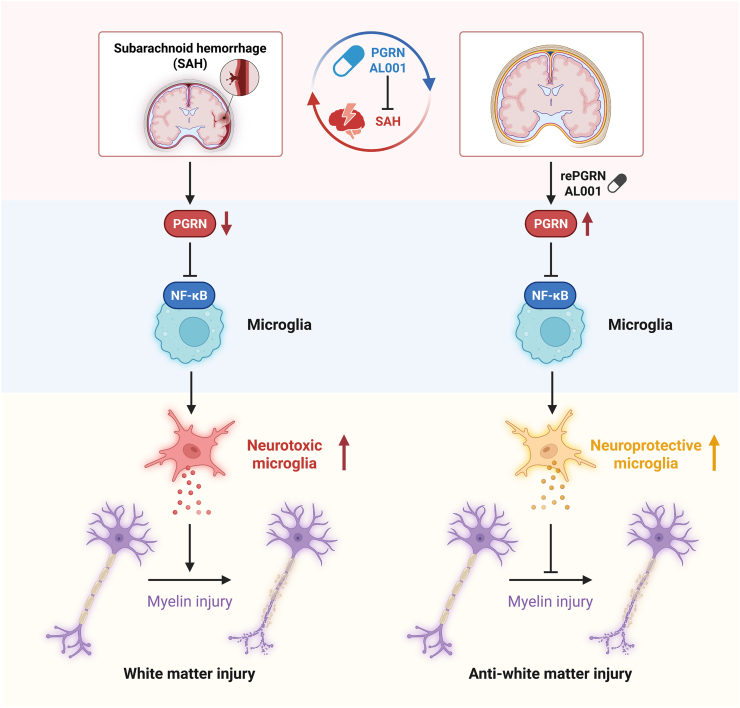
The mechanism diagram of PGRN promoting myelin integrity after SAH.

Progranulin (PGRN) is a widely expressed glycoprotein with multifunctional growth factor properties, playing a critical role in neuroprotection, inflammation regulation, and tissue repair [[6], [7], [8], [9]]. In the central nervous system (CNS), progranulin (PGRN) is mainly localized in neurons and microglial cells, where it plays a crucial role in modulating neuroinflammatory responses. Through these dual potential mechanisms, PGRN helps to maintain immune homeostasis and reduce secondary brain damage following neurological insults [10]. Our previous research has explored the anti-inflammatory role of PGRN in SAH, demonstrating its beneficial effects on neuroprotection. However, the role of PGRN in myelin regeneration after SAH has not been thoroughly investigated. This study aims to explore the potential role of PGRN in myelin integrity following SAH (Fig. 1). We first measured the levels of IBA-1 and MBP in the cerebrospinal fluid of SAH patients, and further investigated the role of PGRN in a rat SAH model, with a focus on its potential mechanisms in myelin integrity and neuroinflammation.

## Materials and Methods

### Experimental procedure

This study consists of five main parts: First, clinical data were collected from patients with trigeminal neuralgia and SAH, including magnetic resonance imaging (MRI) images and cerebrospinal fluid (CSF) samples. The CSF samples were used to measure the protein levels of IBA-1 and MBP through ELISA to assess neuroinjury and myelin integrity. Second, in the animal experiments, male SD rats (280–320 g) were subjected to surgical induction of SAH, followed by PGRN intervention. After SAH, the rats were evaluated for brain white matter myelin damage and the role of PGRN through behavioral tests, immunohistochemistry, Western blotting, and electron microscopy. Third, SAH rats were randomly assigned to either the sham surgery group (Sham) or the SAH group. Brain tissues were collected for transcriptomic sequencing to analyze gene expression changes after SAH, particularly focusing on gene pathways related to glial cell function and myelin integrity. Next, the potential mechanism of PGRN regulating microglia activation through the NF-κB signaling pathway was studied. The effects of PGRN on microglial activation and its role in repairing brain white matter damage after SAH were explored using Western blot, immunofluorescence, and real-time quantitative PCR. Finally, the improvement of cognitive function after AL001 administration was observed through the Morris water maze test, and the damage and repair of brain tissue and myelin were analyzed using MRI, immunohistochemistry, and Western blot.

### Clinical patients

The study included 10 patients with trigeminal neuralgia (control group) and 40 patients with SAH (Table 1). All participants were inpatients at the Department of Neurosurgery, The First Affiliated Hospital of Ningbo University, and were diagnosed based on standard criteria using MRI or angiography. Participants with a history of cardiovascular disease, severe liver disease, or kidney disease were excluded. Diagnoses were independently made by at least two neurosurgeons. Upon hospital admission, the clinical severity of each SAH patient was promptly evaluated using several standardized assessment tools, including the Hunt & Hess grading scale, the modified Fisher scale, the World Federation of Neurosurgical Societies (WFNS) grading system, and the Glasgow Coma Scale (GCS), to ensure a comprehensive appraisal of neurological status. CSF samples were obtained either via lumbar puncture or intraoperatively, depending on the clinical context. The collected CSF was immediately subjected to centrifugation at 3200×*g* for 15 min to separate the supernatant, which was then aliquoted and preserved at −80 °C for subsequent analysis. To evaluate neurological recovery and functional outcomes, the Glasgow Outcome Scale (GOS) and the modified Rankin Scale (mRS) were employed at the time of hospital discharge. All clinical data, including demographic and diagnostic details, are summarized in Table 1, and informed consent was obtained.

**Table 1:** ACA anterior cerebral artery, ACOA anterior communicating artery, ICA internal carotid artery, MCA middle cerebral artery, PCOA posterior communicating artery, BT basilar artery, WFNS World Federation of Neurological Surgeons Grading System, GCS Glasgow Coma Score, GOS Glasgow Outcome Scale, MRS Modified Rankin Scale.

Case no	Age (year)/sex	Aneurysm location	Hunt&Hess grade	Fisher grade	WFNS	GCS	GOS	MRS
1	54/M	LACA	2	–	2	11	3	3
2	67/M	LACA	5	–	4	3	3	5
3	59/F	ACOA	3	4	2	11	3	3
4	65/M	ACOA	2	4	5	3	2	4
5	59/M	LACA	3	–	2	13	3	3
6	61/M	LACA	4	–	5	3	3	4
7	57/F	LMCA	3	2	1	13	3	–
8	50/F	ACOA	3	4	2	11	3	3
9	54/F	LMCA	4	4	2	11	4	4
10	67/M	LMCA	5	4	4	3	1	5
11	69/F	LICA	4	3	4	4	2	3
12	61/F	RPCOA	2	2	2	13	5	1
13	57/M	LMCA	4	4	4	4	2	4
14	44/M	LMCA	2	2	1	15	5	1
15	61/F	LICA	4	3	2	9	3	4
16	78/F	ACOA	1	2	1	15	5	0
17	71/F	LICA	5	2	4	6	5	1
18	67/F	RPCOA	2	3	1	15	4	–
19	76/F	ACOA	5	3	4	3	2	1
20	47/M	RPCOA	3	2	2	15	4	2
21	52/F	ACOA	3	3	1	15	4	4
22	68/F	ACOA	2	3	1	15	5	0
23	58/M	ACOA	4	4	4	3	2	4
24	55/F	RMCA	2	3	2	13	5	1
25	49/M	RICA	5	4	5	3	2	5
26	53/F	LICA	2	4	2	15	5	0
27	56/F	LPCOA	3	2	2	15	5	–
28	60/M	ACOA	2	2	2	14	4	2
29	59/M	RICA	4	4	5	3	2	5
30	47/M	ACOA	2	2	2	14	4	2
31	55/M	ACOA	2	2	2	15	4	3
32	49/F	LICA	2	2	1	15	5	0
33	45/F	ACOA	4	2	1	15	5	0
34	49/M	LACA	4	–	5	3	2	4
35	50/F	RMCA	3	2	3	13	3	4
36	59/F	ACOA	4	4	5	3	1	–
37	37/F	BT	2	3	2	8	3	2
38	58/F	RMCA	3	4	4	3	2	3
39	69/M	ACOA	4	4	5	3	2	4
40	55/M	RMCA	2	2	2	13	4	4
41	48/F	–	–	–	–	–	–	–
42	39/M	–	–	–	–	–	–	–
43	56/M	–	–	–	–	–	–	–
44	48/M	–	–	–	–	–	–	–
45	61/F	–	–	–	–	–	–	–
46	52/M	–	–	–	–	–	–	–
47	59/M	–	–	–	–	–	–	–
48	65/F	–	–	–	–	–	–	–
49	49/M	–	–	–	–	–	–	–
50	46/F	–	–	–	–	–	–	–

### Animal preparation

Male Sprague-Dawley (SD) rats (280 g–320 g), were obtained from the Animal Center of Ningbo University for use in this study. Prior to the commencement of experimental procedures, all animals were housed under standardized and controlled environmental conditions designed to minimize stress and ensure optimal health. These conditions included a maintained ambient temperature of 25 ± 1 °C, a relative humidity range of 60%–70%, and a consistent 12-h light/dark cycle. To allow for physiological and behavioral acclimatization, the rats were permitted to adapt to their environment for no less than 7 days before any interventions. Throughout this adaptation period, they were granted unrestricted access to clean drinking water and standard laboratory chow. Additionally, comprehensive health assessments were performed to screen for any signs of illness, malnutrition, or abnormal behavior; animals failing to meet health criteria were excluded from the study. Approval for the study was granted by the Animal Experiment Ethics Committee of Ningbo University, ensuring that all procedures met institutional and national ethical requirements for animal welfare. Before the experiments, all rats underwent acclimatization training to reduce stress during the procedures. The rats were randomly assigned to different experimental groups to ensure balanced groupings. Throughout the experiment, all rats were handled by trained researchers to avoid unnecessary interference and harm.

### SAH animal model

The experimental SAH model in rats was established using the widely accepted intravascular perforation technique, as previously documented [11]. To initiate the procedure, rats were anesthetized with isoflurane—administered at a concentration of 5% for induction and maintained at 1–2% during the surgical process. Following successful anesthesia, the animals were intubated and positioned supine, after which they were connected to a rodent ventilator system (REWARD, China) delivering a gas mixture composed of 70% medical air and 30% oxygen to ensure adequate ventilation throughout the procedure. The rats underwent puncture using the filament perforation method. Immediately after the perforation, the filament was withdrawn, and the concentration of isoflurane was adjusted to 1.5% to maintain a lighter plane of anesthesia during recovery. Post-surgery, the rats were removed from the ventilator and transferred to a heated pad to facilitate recovery from anesthesia, with core body temperature carefully maintained at approximately 37.5 °C using a feedback-controlled heating system. As a control, sham-operated rats underwent the same surgical preparation and exposure of vessels but without the arterial puncture, thereby ensuring that no hemorrhage was induced in these animals.

### SAH grading

Following established protocols, the severity of SAH in rats was evaluated post-mortem using a standardized SAH grading system [12]. For this assessment, the basal cistern was anatomically divided into six distinct regions. Each region was assigned a score ranging from 0 to 3, depending on the extent and thickness of the subarachnoid blood clot observed—where 0 indicated no visible blood and 3 represented extensive clotting with significant blood accumulation. The overall SAH severity score for each animal was determined by summing the individual scores from all six regions, resulting in a maximum possible score of 18. To ensure consistency and reliability in the experimental model, rats exhibiting a total SAH score of 8 or lower were deemed to have insufficient hemorrhage and were therefore excluded from subsequent analyses. This scoring method allowed for objective quantification of hemorrhage severity and ensured that only animals with moderate to severe SAH were included in the study cohort.

### Drug administration

Based on previous studies, recombinant human PGRN (r-PGRN) from R&D Systems (Minneapolis, MN, USA) was selected for this study. It was diluted with phosphate-buffered saline (PBS) and administered at a concentration of 30 ng/kg via intracerebroventricular (ICV) injection 30 min post-SAH. PBS was used as the vehicle control group. The dose of PGRN was based on our previous research investigating the neuroprotective effects of PGRN after SAH (12). Short hairpin RNA (shRNA) technology mediated by lentivirus (LV-shPGRN) was used to reduce the expression level of PGRN in the rat brain. The lentiviral vector was provided by Genechem (Shanghai, China) with a viral titer of 1E+8 TU/mL, and the ICV injection was performed 72 h prior to modeling. AL001 was administered via intraperitoneal injection at a dose of 5 mL/kg to evaluate its effects on white matter myelin damage and cognitive function after SAH. The control group received an equal volume of PBS injection.

### Intracerebroventricular injection

For stereotaxic injection, experimental rats were placed in a stereotaxic frame and anesthetized with isoflurane. After anesthesia, the needle was inserted into the left lateral ventricle through the bregma using a Hamilton syringe (Hamilton Company, USA, model 701, 10 μL). The precise coordinates for injection were: AP -1.5 mm, ML +1.5 mm, DV -4.0 mm. The injection was performed slowly over approximately 10 min. After the injection, the needle was withdrawn, and the burr hole was sealed quickly with bone wax to prevent contamination. The wound was then sutured, and the animals were awakened from anesthesia and placed in a warm environment for recovery.

### Morris water maze behavioral test

The Morris water maze test was tinted with non-toxic black ink, and the temperature was kept at 23 ± 2 °C. The pool was divided into four quadrants, with a hidden platform (8 cm in diameter, submerged 1.5 cm) positioned in one of the target quadrants, surrounded by spatial cues. To acclimate the animals to the testing environment, all rats were brought into the behavioral testing room 1 h prior to the start of training. The Morris Water Maze training protocol was conducted over a period of four consecutive days, during which each rat underwent four training trials per day. On the fifth day, a spatial memory probe test was carried out to assess memory retention. During the training phase, each rat was randomly released from one of the three quadrants not containing the hidden platform. They were given a maximum of 60 s to freely swim and locate the submerged platform. The escape latency (the time taken to reach the platform) and the swimming path (distance traveled) were automatically recorded and analyzed using AnyMaze behavioral tracking software (Stoelting Co., USA), providing objective performance measurements. On the fifth day, the platform was removed to conduct the probe test. The amount of time spent and the number of times the rat crossed into the former platform quadrant were recorded as indicators of spatial memory and learning ability.

### ELISA

To measure the protein expression levels of IBA-1 and MBP, a gender- and age-matched group of 10 trigeminal neuralgia patients and 40 SAH patients was selected. The cell supernatant was used to measure IL-6 and TNF-α expression. ELISA kits, following the manufacturer's instructions, were used to measure IBA-1 protein levels (MULTISCISCIENCES, Hangzhou, China) and MBP (E-EL-H6156, Elabscience, Wuhan, China). The assays were conducted according to the manufacturer's protocols. The absorbance at 450 nm was measured using a multimode microplate reader (Molecular Devices, USA). Quantitative analysis was performed based on standard curves. Data analysis was performed using the corresponding software.

### Western blot

Equal protein amounts were loaded onto a 4–20% SDS-PAGE gel for electrophoresis and subsequently transferred to a nitrocellulose membrane. The membrane was blocked with 5% non-fat milk at 37 °C for 1.5 h and incubated with the following primary antibodies at 4 °C overnight: anti-PGRN (1:1000, Abcam,USA), anti-MBP (1:1000, Abcam,USA), anti-IBA-1 (1:1000, Abcam,USA), anti-p–NF–kB (1:1000, Cell Signaling Technology,USA), anti–NF–kB (1:1000, Cell Signaling Technology,USA), anti-Arg-1 (1:1000, Thermo Fisher Scientific,USA), anti-iNOS (1:1000, Thermo Fisher Scientific,USA), and anti-β-actin (1:5000, Santa Cruz Biotechnology,USA). The membrane was then incubated with the appropriate horseradish peroxidase-conjugated secondary antibody (1:5000, Abcam,USA) at 37 °C for 1–1.5 h. Protein bands were visualized using the ECL Plus chemiluminescent detection kit (New Cell & Molecular Biotech, China) and quantified using ImageJ software (ImageJ 1.5, NIH, USA).

### Immunofluorescence

The rats were deeply anesthetized, and the entire brain tissue was quickly harvested. The brain was fixed in formalin solution and dehydrated in sucrose solution. After embedding, the brain tissue was sectioned into 10 μm slices using a cryostat. Following membrane permeabilization with 0.3% Triton X-100, the sections were blocked with 5% bovine serum albumin (BSA) and then incubated overnight at 4 °C with the following primary antibodies: anti-myelin basic protein (MBP, 1:200, Abcam, USA), anti-ionized calcium-binding adaptor molecule 1 (IBA-1, 1:200, Abcam, USA), anti-arginase-1 (Arg-1, 1:200, Thermo Fisher Scientific, USA), and anti-interleukin-1β (IL-1β, 1:200, Abcam, USA). After primary antibody incubation, the slides were exposed to fluorophore-conjugated secondary antibodies (1:500, Thermo Fisher Scientific, USA) at room temperature for 1 h in the dark. Immunofluorescence signals were observed and captured using a confocal laser scanning microscope (Leica, Germany). Quantitative analysis of MBP fluorescence was performed in the left corpus callosum using randomly selected coronal sections at 20 × magnification. To evaluate neuroinflammatory responses, the number of IBA-1/Arg-1 and IBA-1/IL-1β double-positive microglia was counted in the left striatum using images taken at 40 × magnification. Fluorescence intensity and cell quantification were carried out with ImageJ software (version 1.5, NIH, USA).

### Magnetic resonance imaging

Following anesthesia, rats were fixed onto a dedicated animal scanning bed, and their respiration and heart rate were monitored to minimize motion artifacts. Brain MRI scans were performed using a 3.0T MRI system (Siemens, Germany) with T2-weighted imaging (T2WI) to assess brain edema and the volume of brain injury. The scanning parameters were as follows: Echo Time (TE): 90 ms, Repetition Time (TR): 4000 ms, Slice Thickness: 2 mm, Field of View (FOV): 60 × 60 mm^2^, Matrix: 256 × 256. After data acquisition, image analysis was conducted using ITK-SNAP (ITK-SNAP, University of Pennsylvania, USA), and the brain injury volume was measured and compared across groups.

### Transmission electron microscopy

Brain tissue for transmission electron microscopy (TEM) was prepared as described in previous reports. Briefly, rats were anesthetized with 5% isoflurane and underwent cardiac perfusion with PBS and 2.5% glutaraldehyde (GA, Sigma). After collecting the tissue samples, they were fixed overnight in 2.5% glutaraldehyde (GA), followed by post-fixation in 1% osmium tetroxide solution for 1–1.5 h. The samples were dehydrated through a graded ethanol series for 10–15 min each, then further dehydrated in 100% ethanol for 15–20 min, and finally in 100% acetone for 15–20 min. The embedding agent was used in a 1:1 mixture of acetone and embedding agent for 40 min to 1 h for infiltration. The process was then continued with a 3:1 mixture of acetone and embedding agent for 2–3 h. The samples were transferred to new centrifuge tubes and incubated with pure embedding agent overnight. The samples were then embedded in freshly prepared Spurr's embedding resin, with the tissue facing downward at the bottom of the embedding tube, along with a labeled pencil mark. The tubes were placed in an oven at 70 °C for polymerization for 24 h or more. Ultra-thin sections were cut using an ultramicrotome to obtain 70–90 nm sections. After staining, images were acquired using a scanning electron microscope (Zeiss Gemini 300). The number of myelinated fibers in the rat striatum was quantified using ImageJ software.

### Statistical analysis

Data analysis was carried out using GraphPad Prism 9 (GraphPad Software, San Diego, CA, USA). Results are presented as mean values with standard deviations (mean ± SD). To compare differences across multiple groups, a one-way analysis of variance (ANOVA) was conducted, followed by Tukey's post hoc test for multiple comparisons. Long-term neurobehavioral data were assessed using two-way ANOVA. A p-value less than 0.05 was regarded as indicative of statistical significance.

## Result

### Expression of PGRN, IBA-1, and MBP levels and white matter injury in clinical SAH patients

**Fig. 2:**
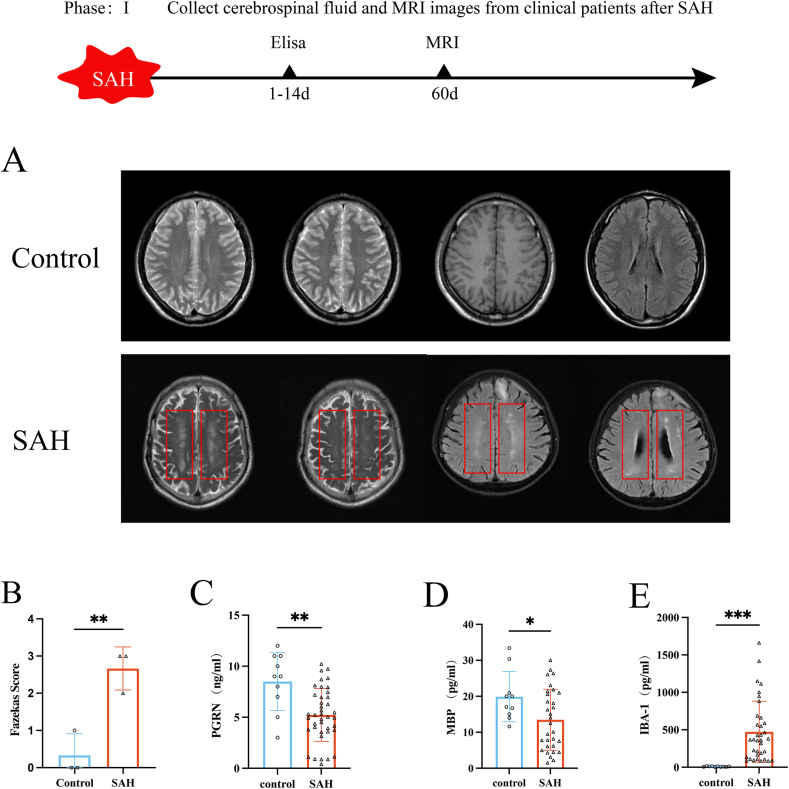
MRI results of SAH patients and changes in PGRN, IBA-1, and MBP levels in cerebrospinal fluid:(A) MRI images of SAH patients 60 days post-follow-up. (B) MRI images showing the Fazekas score of the white matter region in SAH patients, n = 3. (C–E) Comparison of the levels of PGRN, IBA-1, and MBP in cerebrospinal fluid between the control group and SAH patients, with the control group n = 10 and SAH group n = 40. ∗*P <* 0.05, ∗∗*P <* 0.01, ∗∗∗*P <* 0.001.

We followed up with three SAH patients two months after discharge. MRI analysis revealed that white matter abnormalities were more pronounced in patients with subarachnoid hemorrhage (SAH) than in those from the control group, as reflected by significantly elevated Fazekas scores (*P <* 0.01; see Fig. 2A and B). To further investigate the molecular changes associated with SAH, CSF samples were obtained from a total of 50 individuals. Among them, 40 were diagnosed with SAH, while the remaining 10, serving as controls, were patients diagnosed with trigeminal neuralgia who required lumbar puncture for clinical reasons. The expression levels of PGRN, IBA-1, and MBP in the CSF were quantified using ELISA. Results demonstrated a marked decrease in PGRN concentration in the CSF of SAH patients when compared to the control group (*P <* 0.01; Fig. 2C). Conversely, the levels of IBA-1, a marker of microglial activation, were significantly elevated in the SAH group (*P <* 0.001; Fig. 2D). In addition, MBP levels, which are indicative of myelin integrity, were found to be significantly reduced in SAH patients relative to controls (*P <* 0.05; Fig. 2E), suggesting potential white matter damage associated with the condition. These findings suggest that white matter injury is more pronounced in SAH patients, possibly indicating microvascular changes or inflammation-related demyelination. The decreased PGRN levels could suggest reduced neuroprotective effects, and the reduction of PGRN, as an anti-inflammatory factor, may have exacerbated the inflammatory response. The elevated IBA-1 levels suggest that the underlying potential mechanism may involve inflammation imbalance, microglial overactivation, and demyelination caused by the decline in PGRN.

### Myelin damage levels after PGRN injection into the lateral ventricle in rats

**Fig. 3:**
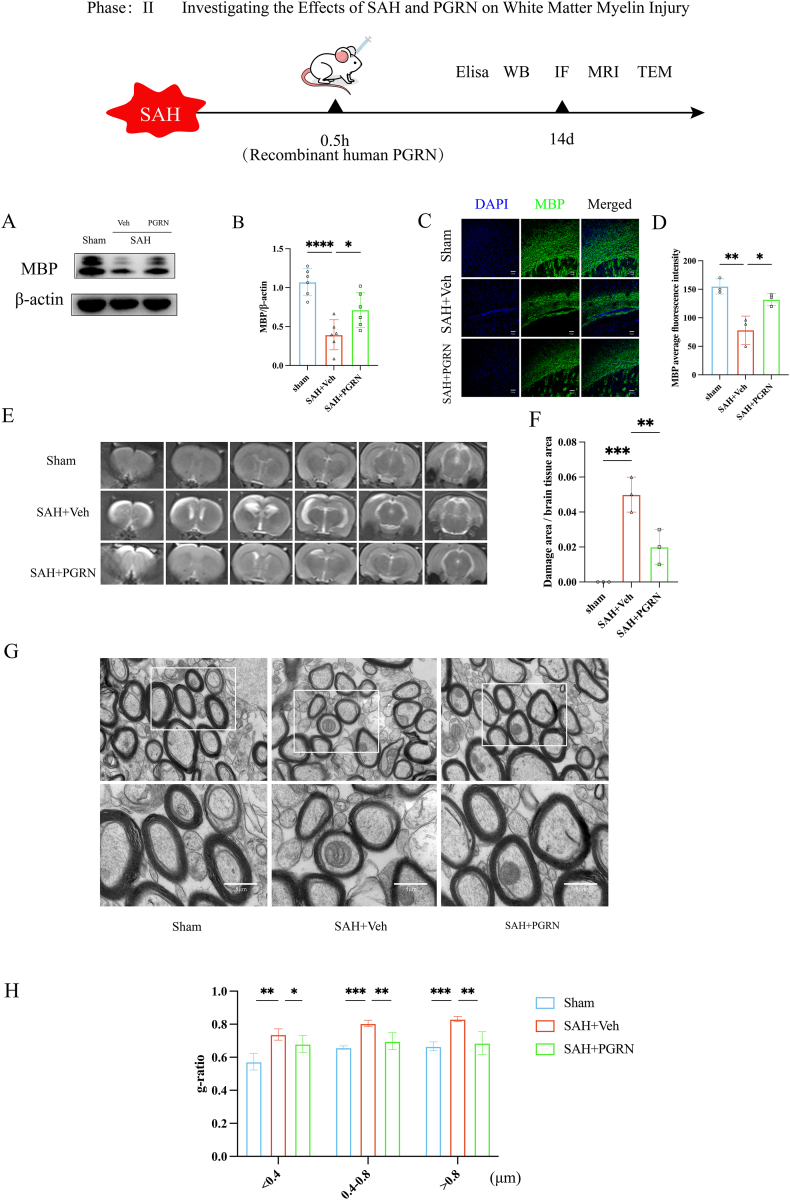
The role of PGRN in white matter myelin integrity in SAH rats: (A–B) Expression of MBP in rat brain tissue, n = 6. (C–D) MBP staining of the corpus callosum to observe the degree of myelin injury in three groups of rats, scale bar 50 μm, n = 3. (E–F) MRI scans of brain tissue in three groups of rats, with ITK-SNAP analysis of brain lesion areas, n = 3. (G) Electron microscopy showing myelin sheath thickness and g-ratio (inner diameter of myelin/outer diameter of myelin). (H) Evaluation of myelin injury degree using g-ratio for different myelin diameters (<0.4 μm, 0.4–0.8 μm, >0.8 μm), n = 3, 200–400 myelin sheaths per rat. ∗*P <* 0.05, ∗∗*P <* 0.01, ∗∗∗*P <* 0.001.

Rat brain tissue from the left hemisphere was analyzed by Western blot to measure MBP expression after SAH. The experimental results demonstrated a marked decrease in myelin basic protein (MBP) expression in the SAH group when compared to the sham-operated group, as revealed by Western blot analysis (*P <* 0.01; Fig. 3A and B), indicating significant myelin damage following subarachnoid hemorrhage. However, administration of progranulin (PGRN) led to a substantial increase in MBP levels in the SAH group relative to the untreated SAH animals (*P <* 0.05; Fig. 3A and B), suggesting that PGRN may exert a protective effect on myelin integrity. Similarly, immunofluorescence staining supported these findings, showing a notable reduction in MBP fluorescence intensity in the SAH group compared to the sham controls (*P <* 0.01; Fig. 3C and D). In contrast, rats treated with PGRN displayed a visibly enhanced MBP signal (*P <* 0.05; Fig. 3C and D), indicating that PGRN supplementation helps to alleviate myelin degradation. These data collectively reinforce the hypothesis that SAH induces white matter injury and that PGRN may promote myelin preservation and repair in the damaged brain regions.

MRI analysis showed that the lesion area in the SAH group was significantly larger than in the sham group (*P <* 0.001, Fig. 3E and F). However, the PGRN-treated SAH group showed less brain damage than the untreated SAH group (*P <* 0.01, Fig. 3E and F), with the hippocampus being the main site of injury. After euthanizing these rats, electron microscopy was performed to observe the myelin damage. To quantitatively assess the extent of myelin damage, the g-ratio was utilized as an indicator, defined as the ratio of the axonal diameter to the total diameter including the myelin sheath. An elevated g-ratio typically reflects thinner myelin and, consequently, more pronounced demyelination. Transmission electron microscopy revealed that in the SAH + vehicle (Veh) group, the myelin sheaths were significantly thinner than those in the sham-operated group, as evidenced by the increased g-ratio values (Fig. 3G), suggesting substantial myelin disruption following SAH. Upon administration of exogenous progranulin (PGRN), the myelin structure showed marked improvement, with thicker myelin sheaths and a corresponding reduction in g-ratio values (Fig. 3G), indicating a protective or restorative effect of PGRN on white matter integrity. Further stratified analysis based on the diameter of myelinated axons (<0.4 μm, 0.4–0.8 μm, and >0.8 μm) demonstrated that the SAH + Veh group consistently exhibited higher g-ratio values across all diameter ranges when compared to the sham group, indicating widespread myelin thinning (<0.4 μm: *P <* 0.01; 0.4–0.8 μm: *P <* 0.001; >0.8 μm: *P <* 0.001; Fig. 3H). In contrast, the rats treated with PGRN showed significantly lower g-ratios across these same diameter categories (<0.4 μm: *P <* 0.05; 0.4–0.8 μm: *P <* 0.01; >0.8 μm: *P <* 0.01; Fig. 3H), further supporting the conclusion that PGRN treatment can effectively mitigate myelin damage induced by SAH. These results suggest that SAH causes significant myelin damage, as evidenced by decreased MBP expression, increased lesion size on MRI, and a higher g-ratio (thinner myelin). PGRN treatment promotes myelin integrity and neuroprotection by increasing MBP expression, reducing brain damage area, and lowering the g-ratio. These findings suggest that PGRN has the potential to be developed into a therapeutic strategy aimed at restoring myelin structure and function in the context of SAH-induced white matter injury.

### Myelin damage levels after knockdown of PGRN in rats

**Fig. 4:**
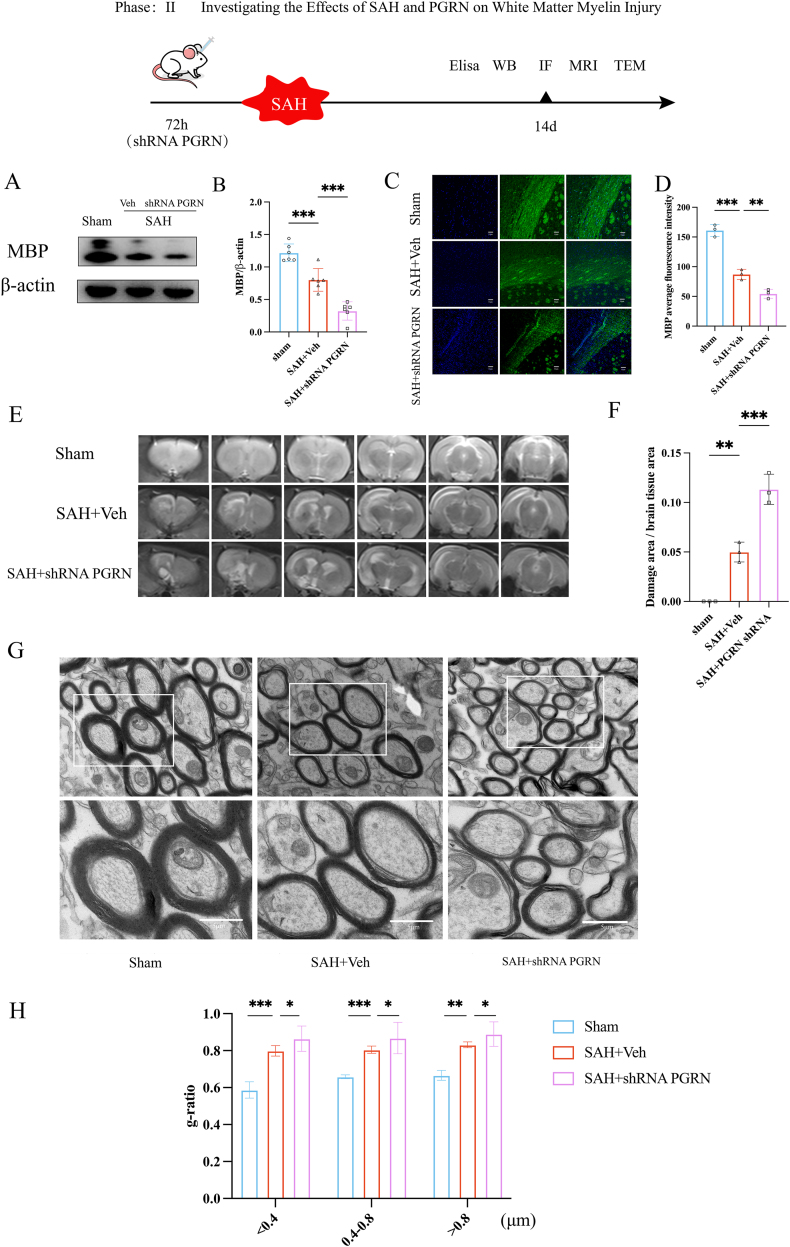
The effect of PGRN knockdown on white matter myelin integrity in SAH rats: (A–B) Expression of MBP in rat brain tissue, n = 6. (C–D) MBP staining of the corpus callosum to observe the degree of myelin injury in three groups of rats, scale bar 50 μm, n = 3. (E–F) MRI scans of brain tissue in three groups of rats, with ITK-SNAP analysis of brain lesion areas, n = 3. (G) Electron microscopy showing myelin sheath thickness and g-ratio (inner diameter of myelin/outer diameter of myelin). (H) Evaluation of myelin injury degree using g-ratio for different myelin diameters (<0.4 μm, 0.4–0.8 μm, >0.8 μm), n = 3, 200–400 myelin sheaths per rat. ∗*P <* 0.05, ∗∗*P <* 0.01, ∗∗∗*P <* 0.001.

To further investigate the role of PGRN in the rat brain, we used lentivirus transfection to suppress PGRN expression. Western blot analysis was performed on brain tissue from the left hemisphere of rats to measure MBP expression after SAH. The experimental data demonstrated that myelin basic protein (MBP) expression was markedly reduced in the SAH group when compared to the sham-operated controls (*P <* 0.05, Fig. 4A and B). This reduction became even more pronounced following the knockdown of PGRN, with MBP levels in the PGRN-deficient SAH group significantly lower than those in the SAH group without genetic intervention (*P <* 0.05, Fig. 4A and B). Consistent with the Western blot findings, immunofluorescence analysis further revealed a substantial decrease in MBP expression in the SAH group relative to the sham group (*P <* 0.001, Fig. 4C and D). Notably, the group subjected to PGRN knockdown exhibited a near-complete loss of MBP signal (*P <* 0.01, Fig. 4C and D), indicating severe myelin impairment under conditions of PGRN deficiency.

MRI analysis revealed that the lesion area in the SAH group with PGRN knockdown was larger and the damage more severe compared to the untreated SAH group (*P <* 0.001, Fig. 4E and F). After euthanizing the rats, electron microscopy of brain tissue showed that the myelin sheath was significantly thinner in the PGRN knockdown group compared to the SAH + Veh group (Fig. 4G). In the comparison of myelin sheath diameters (<0.4 μm, 0.4–0.8 μm, >0.8 μm), we found that the g-ratio was higher in the SAH + Veh group compared to the sham group (<0.4 μm: *P <* 0.001; 0.4–0.8 μm: *P <* 0.001; >0.8 μm: *P <* 0.01, Fig. 4H). After PGRN knockdown, the g-ratio in the SAH group increased even further compared to the SAH + Veh group (<0.4 μm: *P <* 0.05; 0.4–0.8 μm: *P <* 0.05; >0.8 μm: *P <* 0.05, Fig. 4H). These results suggest that PGRN not only alleviates myelin damage after SAH but also that its absence exacerbates myelin loss and neural damage, highlighting PGRN as an important regulator of myelin integrity following SAH.

### Transcriptome sequencing

**Fig. 5:**
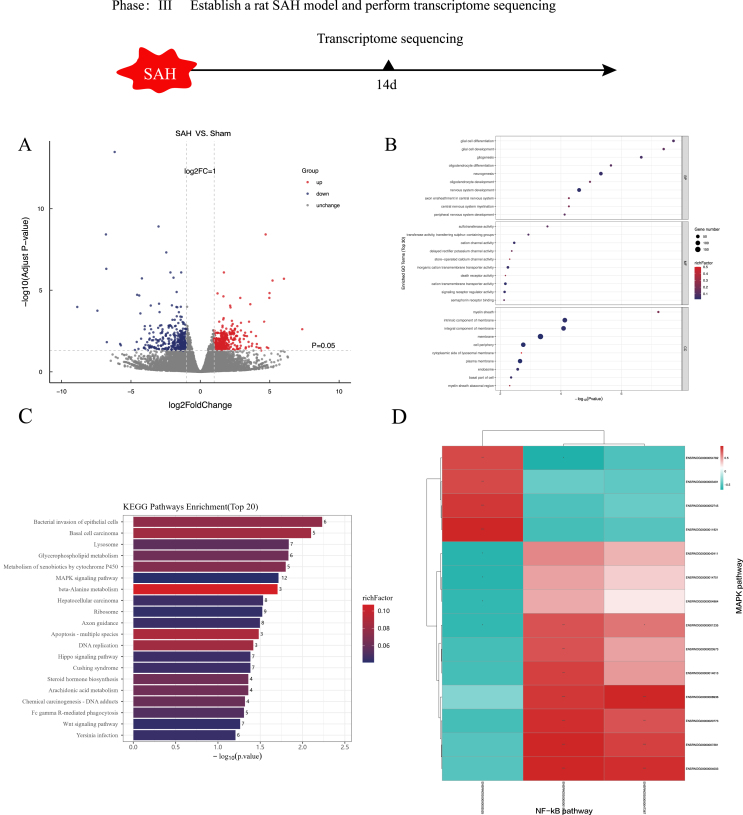
Transcriptome sequencing analysis results:(A) Differential genes identified in rat brain tissue after transcriptome sequencing of Sham and SAH groups. (B) Results of GO enrichment analysis, showing the top 10 functional terms in biological processes (BP), molecular functions (MF), and cellular components (CC) categories. (C) Results of KEGG pathway enrichment analysis, showing the main signaling pathways enriched in differential genes. (D) Correlation analysis of NF-κB signaling pathway-related genes and MAPK signaling pathway-related genes. ∗*P <* 0.05, ∗∗*P <* 0.01, ∗∗∗*P <* 0.001.

To further investigate the specific potential mechanisms through which PGRN mitigates myelin damage following SAH, we performed transcriptome sequencing on brain tissue from the Sham and SAH groups. A total of 683 differentially expressed genes (DEGs) were identified through transcriptomic analysis, comprising 346 genes that were significantly upregulated and 337 that were downregulated (Fig. 5A). To gain insights into the potential biological roles of these DEGs, Gene Ontology (GO) enrichment analysis was performed. The analysis revealed the top 10 significantly enriched functional terms across each of the three main GO categories—Biological Process (BP), Molecular Function (MF), and Cellular Component (CC)—resulting in a total of 30 enriched GO terms. Within the BP category, the most prominent biological processes associated with the DEGs included glial cell differentiation, glial cell development, gliogenesis, CNS development, and peripheral nervous system (PNS) development, indicating a strong involvement in neurodevelopmental and glial-related pathways. MF (Molecular Function): The top enriched terms included 2'−5'−oligoadenylate synthetase activity, sulfotransferase activity, transporter activity, transmembrane transporter activity, and transferase activity. CC (Cellular Component): The main enriched terms were myelin sheath, Schmidt−Lanterman incisure, myelin sheath adaxonal region, compact myelin, and basal part of cell (Fig. 5B).

In KEGG pathway enrichment analysis, the differentially expressed genes were primarily enriched in pathways such as bacterial invasion of epithelial cells, COVID-19, Hippo signaling pathway, oxidative phosphorylation, and MAPK signaling pathway (Fig. 5C).

We specifically focused on the NF-κB signaling pathway, but no significant differential expression of related genes was observed in the transcriptome sequencing. To further explore the potential role of the NF-κB pathway, we conducted a correlation analysis between three NF-κB-related genes and 14 MAPK-related genes. The results revealed a positive correlation between most of the genes (Fig. 5D). These findings imply a potential crosstalk between the MAPK signaling pathway and the NF-κB pathway, suggesting that MAPK signaling may be intricately involved in the regulatory potential mechanisms underlying PGRN-mediated myelin integrity after subarachnoid hemorrhage (SAH). The MAPK pathway could therefore serve as a critical downstream or parallel effector contributing to the protective and restorative effects of PGRN on white matter integrity following SAH-induced injury.

### PGRN protects brain neurons by regulating microglial activation

**Fig. 6:**
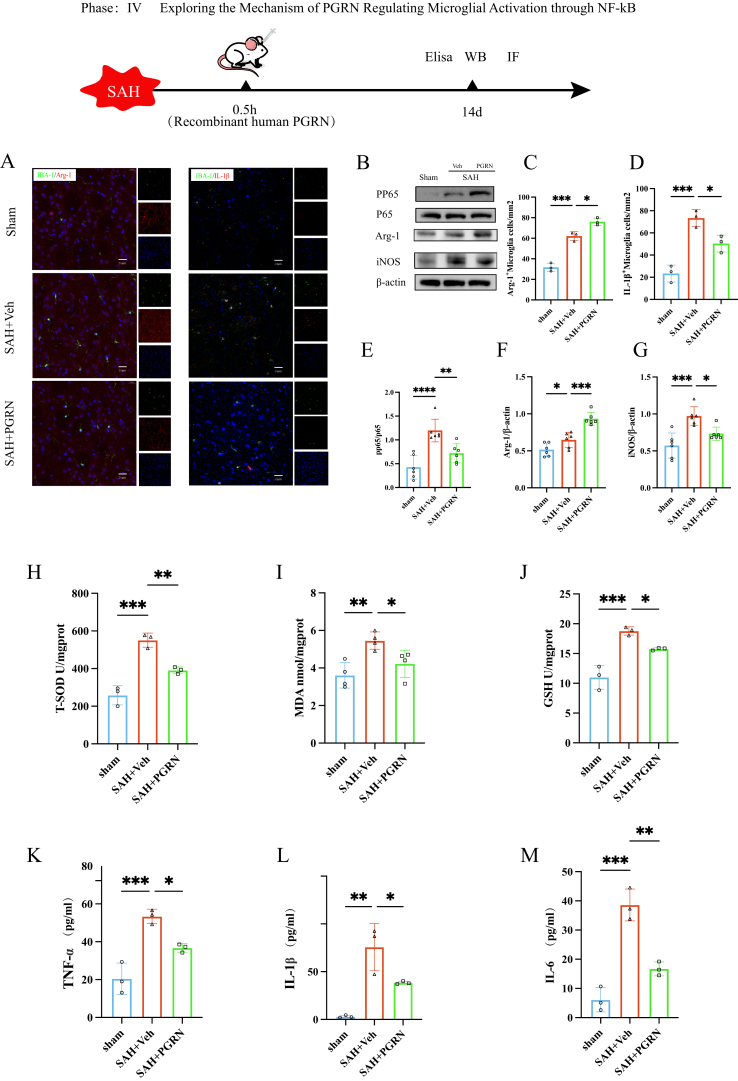
The effect of PGRN on microglial activation, NF-κB pathway activation, and oxidative stress after SAH:(A) Immunofluorescence images of co-staining Arg-1 and IL-1β with IBA-1. (B) Western blot images detecting NF-κB, Arg-1, and iNOS expression. (C–D) Number of positive cells for Arg-1 and IL-1β, n = 3. (E–G) Western blot analysis of NF-κB, Arg-1, and iNOS expression in the SAH group and PGRN treatment group, n = 6. (H–J) Evaluation of PGRN's effect on oxidative stress by measuring MDA, T-SOD, and GSH levels, n = 3. (K–M) ELISA analysis of the expression changes of inflammatory factors (TNF-α, IL-1β, IL-6, IL-10) to assess the modulation of inflammation by PGRN, n = 3. ∗*P <* 0.05, ∗∗*P <* 0.01, ∗∗∗*P <* 0.001.

Although the NF-κB pathway was not directly enriched in the transcriptome analysis, the genes related to NF-κB showed a positive correlation with MAPK signaling pathway genes. The MAPK signaling pathway is closely linked to inflammation and the activation of microglia, suggesting that PGRN may affect microglial activation through the NF-κB/MAPK axis. GO analysis indicated that biological processes (BP) were enriched in glial cell differentiation, nervous system development, and other functions related to microglial activation and myelin integrity. Cellular component (CC) analysis highlighted myelin and glial cell structures, further supporting the role of PGRN in the CNS inflammatory environment. To further verify the relationship between PGRN and microglial activation after SAH, IL-1β and iNOS were selected as representative pro-inflammatory activation markers, while Arg-1 was used as a marker associated with anti-inflammatory or reparative microglial responses. We acknowledge that these markers represent only a subset of microglial activation states rather than discrete M1/M2 phenotypes. IBA-1 staining was used to evaluate changes in microglial activation profiles following PGRN overexpression. The quantitative analysis revealed that, relative to the Sham group, the SAH + Veh group exhibited a markedly reduced number of Arg-1-positive microglia, suggesting a shift in microglial inflammatory marker expression following SAH. (*P <* 0.001, Fig. 6A and C). Notably, overexpression of PGRN significantly increased the number of Arg-1–positive microglia in the SAH + PGRN group compared with the SAH + Veh group (P < 0.05, Fig. 6A and C), indicating that PGRN may promote microglial states associated with anti-inflammatory marker expression. In contrast, the number of IL-1β–positive microglia, reflecting a pro-inflammatory activation state, was significantly elevated in the SAH + Veh group compared with Sham controls (P < 0.001, Fig. 6A and D). This increase was markedly attenuated in the SAH + PGRN group (P < 0.05, Fig. 6A and D). Collectively, these findings suggest that PGRN modulates microglial inflammatory activation following SAH.

Furthermore, we focused on the role of PGRN in NF-κB activation. It was postulated that PGRN may regulate microglial activation through modulation of the NF-κB signaling pathway, thereby influencing neuroinflammation and oxidative stress, ultimately impacting neuronal injury and subsequent recovery following SAH. To explore this hypothesis, Western blot analysis was employed to assess the activation of the NF-κB pathway (via phosphorylated p65, p-p65) and to quantify the expression levels of activation-associated markers, iNOS and Arg-1. The findings demonstrated that NF-κB pathway activation was significantly enhanced in the SAH + Veh group relative to the Sham group (*P <* 0.0001; Fig. 6B and E), indicating a robust inflammatory response. Notably, administration of PGRN markedly suppressed p-p65 expression (*P <* 0.01; Fig. 6B and E), suggesting that PGRN may influence NF-κB signaling activity. In terms of activation markers, Arg-1 expression showed a modest yet significant increase in the SAH + Veh group when compared to Sham controls (*P <* 0.05; Fig. 6B and F), and this increase was further amplified following PGRN treatment (*P <* 0.001; Fig. 6B and F), Conversely, iNOS levels, were substantially elevated in the SAH + Veh group compared to the Sham group (*P <* 0.001; Fig. 6B and G), while treatment with PGRN led to a notable reduction in iNOS expression (*P <* 0.05; Fig. 6B and G). These results collectively indicate that PGRN treatment was associated with reduced NF-κB activation and altered microglial inflammatory profiles, suggesting that NF-κB signaling may be involved in the observed protective effects.

To further investigate the regulatory role of PGRN in oxidative stress following SAH, we quantified the levels of key oxidative stress biomarkers, including malondialdehyde (MDA), total superoxide dismutase (T-SOD), and glutathione (GSH). As shown in Fig. 6H–J, rats in the SAH + Veh group exhibited a significant increase in oxidative stress markers when compared to the Sham group, with elevated levels of T-SOD (*P <* 0.001), MDA (*P <* 0.01), and GSH (*P <* 0.001), suggesting a heightened oxidative response following hemorrhage. However, overexpression of PGRN led to a notable attenuation of this stress response. Specifically, PGRN treatment significantly reduced the levels of T-SOD (*P <* 0.01), MDA (*P <* 0.05), and GSH (*P <* 0.05) compared to the SAH + Veh group, indicating that PGRN may exert protective effects by modulating the oxidative microenvironment in the brain post-SAH. These findings highlight the potential antioxidative role of PGRN in mitigating secondary injury potential mechanisms associated with SAH. Furthermore, ELISA analysis of inflammatory factors (TNF-α, IL-1β, IL-6, IL-10) revealed that the SAH + Veh group had significantly higher levels of inflammatory factors compared to the Sham group (*P <* 0.001 for TNF-α and IL-6, *P <* 0.01 for IL-1β, Fig. 6K–M). PGRN treatment significantly inhibited these inflammatory responses (*P <* 0.05 for TNF-α and IL-1β, *P <* 0.01 for IL-6, Fig. 6K–M). These findings suggest that PGRN may exert neuroprotective effects after SAH partly through modulation of inflammatory signaling and microglial activation states.

### Knockdown of PGRN reverses inflammatory protective effects after microglial activation

**Fig. 7:**
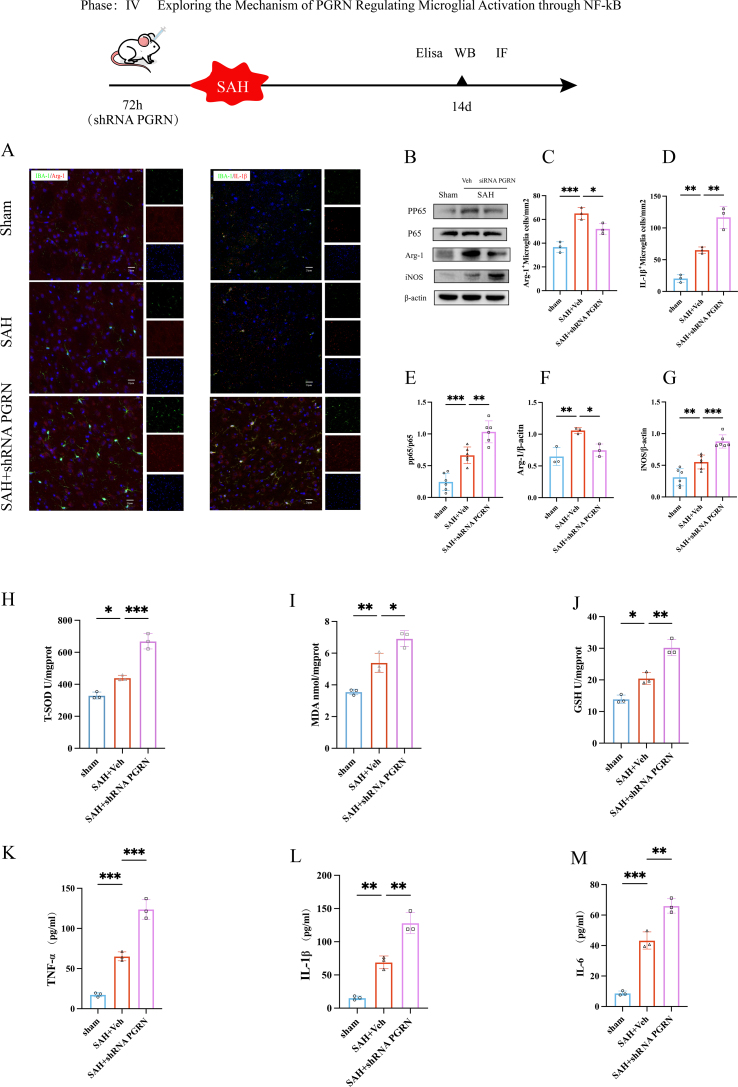
The effect of PGRN knockdown on microglial activation, NF-κB pathway activation, and oxidative stress after SAH:(A) Immunofluorescence images of co-staining Arg-1 and IL-1β with IBA-1. (B) Western blot images detecting NF-κB, Arg-1, and iNOS expression. (C–D) Number of positive cells for Arg-1 and IL-1β, n = 3. (E–G) Western blot analysis of NF-κB, Arg-1, and iNOS expression in the SAH group and PGRN knockdown group, n = 6. (H–J) Evaluation of PGRN's effect on oxidative stress by measuring MDA, T-SOD, and GSH levels, n = 3. (K–M) ELISA analysis of the expression changes of inflammatory factors (TNF-α, IL-1β, IL-6, IL-10) to assess the modulation of inflammation by PGRN, n = 3. ∗P < 0.05, ∗∗P < 0.01, ∗∗∗P < 0.001.

To further verify the role of PGRN in microglial activation and its regulation of the NF-κB signaling pathway after SAH, we knocked down PGRN using a lentiviral shRNA approach and divided the rats into Sham, SAH + Veh, and SAH + shRNA PGRN groups. The same experiments were repeated to explore the impact of PGRN downregulation on inflammation and oxidative stress. Immunofluorescence co-staining results showed that, compared to the SAH + Veh group, PGRN knockdown further reduced Arg-1 (*P <* 0.05, Fig. 7A and C), while the expression of IL-1β was significantly increased (*P <* 0.01, Fig. 7A and D), indicating that PGRN knockdown promoted microglia activation toward a neurotoxic microglia, which may exacerbate neuroinflammation. The results revealed that, compared to the SAH + Veh group, the expression of p-p65 (NF-κB activation marker) was further upregulated after PGRN knockdown (*P <* 0.01, Fig. 7B and E), while Arg-1 expression was further reduced (*P <* 0.05, Fig. 7B and F), and iNOS was significantly elevated (*P <* 0.001, Fig. 7B and G). This suggests that PGRN loss may exacerbate microglial activation through the activation of the NF-κB pathway. Oxidative stress measurements indicated that, compared to the SAH + Veh group, PGRN knockdown led to further increases in MDA levels (*P <* 0.001, Fig. 7H) and significant increases in SOD and GSH levels (*P <* 0.05 for SOD, *P <* 0.01 for GSH, Fig. 7I and J), suggesting that the loss of PGRN exacerbates oxidative damage induced by SAH. ELISA results showed that, compared to the SAH + Veh group, PGRN knockdown led to further increases in the expression of inflammatory cytokines TNF-α, IL-1β, and IL-6 (*P <* 0.001 for TNF-α, *P <* 0.01 for IL-1β, *P <* 0.01 for IL-6, Fig. 7K–M), indicating that PGRN might regulate the inflammatory environment after SAH by inhibiting the NF-κB-mediated pro-inflammatory response. In summary, in contrast to the protective effects of PGRN overexpression, PGRN knockdown exacerbates the activation of the NF-κB signaling pathway, microglial activation, oxidative stress, and neuroinflammation, further supporting the role of PGRN in neuroprotection after SAH, likely by inhibiting NF-κB signaling to promote microglial activation to a protective state and reduce inflammation.

### Effect of PGRN on neurological behavior and spatial memory after SAH

**Fig. 8:**
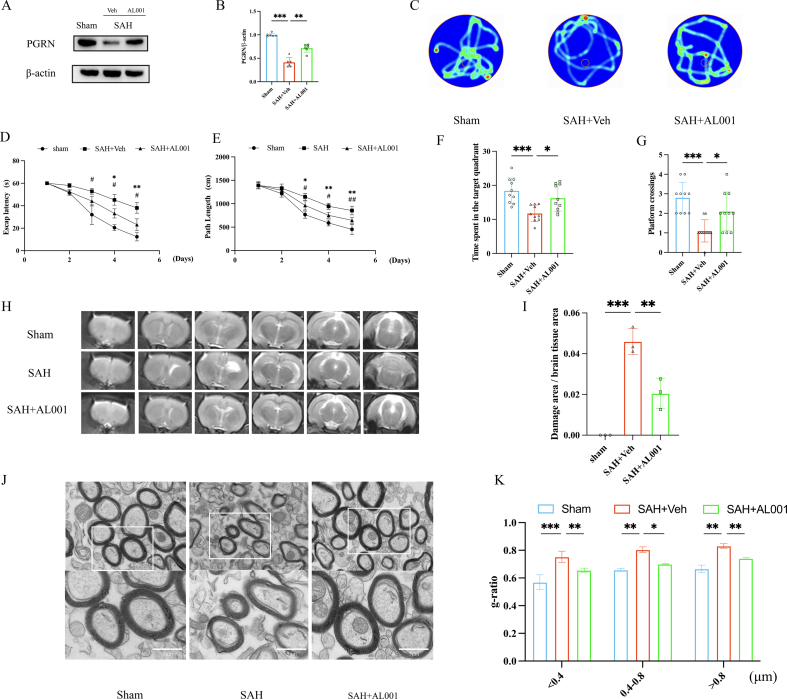
The effect of AL001 in improving neuroinjury after SAH by upregulating PGRN: (A–B) Western blot analysis of PGRN expression levels in the SAH + Veh group and SAH + AL001 group, n = 6. (C–G) Morris water maze experiment assessing cognitive function recovery after SAH, represented by changes in escape latency, target quadrant dwell time, and platform crossing frequency in different groups, n = 3. (H–I) MRI assessment of brain tissue injury in the SAH + AL001 group and SAH + Veh group, showing comparison of lesion volume, n = 3. (J–K) Transmission electron microscopy showing myelin morphology, displaying changes in myelin thickness in the SAH + AL001 group, g-ratio analysis results, and evaluation of myelin injury degree using g-ratio for different myelin diameters (<0.4 μm, 0.4–0.8 μm, >0.8 μm), n = 3, 200–400 myelin sheaths per rat. #/∗*P <* 0.05, ##/∗∗*P <* 0.01, ###/∗∗∗*P <* 0.001.

To further investigate whether increasing PGRN expression could improve SAH-induced neurological damage, we used AL001 (a PGRN expression enhancer) to intervene in the SAH rats and set up Sham, SAH + Veh, and SAH + AL001 groups. We assessed the effects of AL001-mediated upregulation of PGRN at the molecular, behavioral, imaging, and ultrastructural levels. Western blot results showed that, compared to the SAH + Veh group, PGRN expression was significantly increased in the SAH + AL001 group (*P <* 0.01, Fig. 8A and B), confirming the effectiveness of AL001 as a PGRN enhancer. To further examine the effects of AL001 on cognitive impairment after SAH, we performed the Morris water maze test at 4 weeks post-SAH. The results showed that, over the 5-day training period, the Sham group rats had the best learning and memory abilities, while the SAH + Veh group had the weakest. After AL001 treatment, the escape latency of the rats was significantly reduced (Fig. 8C, D, 8E). Compared with the SAH + Veh group, rats treated with AL001 (SAH + AL001 group) demonstrated markedly improved cognitive performance in the Morris water maze test. Specifically, these rats spent significantly more time in the target quadrant (*P <* 0.05, Fig. 8C and F) and exhibited an increased number of platform crossings (*P <* 0.05, Fig. 8C and G), indicating enhanced spatial memory and learning ability. These behavioral improvements imply that AL001 may facilitate cognitive recovery following SAH, potentially through the upregulation of PGRN expression. Moreover, MRI analysis revealed that the SAH + AL001 group exhibited a significantly smaller lesion volume relative to the SAH + Veh group (*P <* 0.01, Fig. 8H and I), suggesting that the neuroprotective effects of PGRN may contribute to mitigating structural brain damage after hemorrhage. Consistent with these findings, transmission electron microscopy of myelin ultrastructure showed that myelin thickness was notably increased in the SAH + AL001 group compared to the SAH + Veh group. Correspondingly, the g-ratio was significantly decreased, indicating improved myelination (g-ratio <0.4 μm: *P <* 0.01; 0.4–0.8 μm: *P <* 0.05; >0.8 μm: *P <* 0.01; Fig. 8J and K). These results collectively suggest that AL001 exerts a beneficial effect on myelin integrity and neurofunctional recovery post-SAH, potentially through enhancing endogenous PGRN signaling.

## Discussion

This study investigated the role of PGRN in microglial activation, neuroinflammation, oxidative stress, and neurological recovery after subarachnoid hemorrhage (SAH). In our preliminary clinical observations, MRI suggested the presence of white matter lesions in SAH patients, and CSF analysis suggested reduced levels of PGRN and MBP, alongside elevated IBA-1 expression after SAH. In the rat model, overexpression of PGRN resulted in higher MBP expression, less brain tissue damage on MRI, and reduced myelin injury, while PGRN knockdown reversed these effects. Transcriptomic sequencing results indicated that SAH primarily affects pathways related to inflammation regulation, oxidative stress, glial cell activation, and myelination. Immunofluorescence and Western blotting results demonstrated that SAH induced abnormal activation of microglia, exacerbating inflammatory responses and oxidative stress., characterized by elevated expression of pro-inflammatory factors such as IL-1β and iNOS. In contrast, PGRN overexpression significantly inhibited the abnormal activation of microglia, promoting their anti-inflammatory and antioxidant neuroprotective functions. These findings indicate that PGRN treatment coincides with attenuation of NF-κB activation and shifts in microglial inflammatory markers; however, whether NF-κB signaling is required for PGRN-mediated effects remains to be determined, reducing neuroinflammation and alleviating secondary damage associated with SAH. Oxidative stress-related biomarkers measured after SAH revealed elevated MDA levels and increased SOD and GSH activity in the SAH group, while PGRN overexpression significantly reduced MDA levels and improved antioxidant enzyme activity. This suggests that PGRN may reduce SAH-induced oxidative damage by enhancing antioxidant capacity. Morris water maze experiments showed that the SAH + Veh group had longer escape latencies, decreased time spent in the target quadrant, and fewer platform crossings, indicating significant cognitive impairment after SAH. In contrast, treatment with the PGRN enhancer AL001 significantly improved the rats' learning and memory abilities. MRI and electron microscopy results indicated that AL001 treatment reduced brain and myelin damage, further supporting the potential neuroprotective effect of PGRN. Our findings suggest that PGRN treatment is associated with reduced NF-κB activation and altered microglial inflammatory profiles after SAH. However, whether NF-κB signaling is required for PGRN-mediated neuroprotection requires further mechanistic investigation. We found that high PGRN expression can improve neurological function and could offer new insights into therapeutic strategies aimed at improving SAH outcomes.

PGRN plays a crucial role in regulating nerve regeneration, and its regulatory effects are observed throughout the entire process of neurogenesis and development [13]. As a growth factor, PGRN promotes cell proliferation and neurovascular growth, and its therapeutic effects on white matter damage have been well-documented in various studies [14]. However, despite its significant role in the brain, the specific function and potential targets of PGRN in white matter myelin damage following subarachnoid hemorrhage (SAH) have not been clearly reported. Our study confirms that PGRN is downregulated after SAH, accompanied by a reduction in MBP, suggesting that it may be involved in the process of myelin injury. Previous research indicates that the absence of PGRN alters microglial function and compromises lysosomal activity, thereby contributing to myelin debris buildup [15], which is consistent with our findings. Additionally, microglia are key players in the inflammatory response within the CNS, and after SAH, they are rapidly polarized to produce inflammatory factors, further exacerbating neuroinflammation and white matter damage [16]. We observed that following SAH, PGRN expression decreased, leading to abnormal activation of microglia and further aggravating brain injury. This suggests that PGRN may regulate microglial inflammatory activation states, influencing both the clearance and repair of myelin. Our findings highlight the potential of PGRN as a therapeutic target for mitigating white matter damage and promoting myelin integrity in the context of SAH. However, it should be noted that the markers used in this study represent only a limited subset of microglial activation states and do not fully capture the complexity and heterogeneity of microglial responses after SAH. Future studies using single-cell approaches or broader marker panels will be required to more comprehensively define microglial phenotypic diversity in this context.

Based on transcriptomic analysis, we further explored the molecular potential mechanisms induced by SAH and found that the SAH group showed significant enrichment in genes related to glial cell differentiation, myelin-associated structures, and immune regulation. This is consistent with the role of PGRN in myelin integrity and neuroprotection [17]. GO enrichment analysis results indicated that SAH primarily affects biological processes such as immune response, myelin formation, cell apoptosis, and inflammatory factor regulation, with pathways related to microglial cell activation and myelin integrity being significantly enriched. Considering that PGRN has properties of regulating microglial activation and promoting neurovascular regeneration, we speculate that PGRN may indirectly affect the white matter damage and repair process induced by SAH by regulating the inflammatory state of microglial cells.

KEGG pathway enrichment analysis indicated that the genes differentially expressed in response to SAH were significantly associated with several key signaling cascades, including the Hippo signaling pathway, oxidative phosphorylation, and the MAPK signaling pathway. Notably, the Hippo pathway is known to play a crucial role in modulating various aspects of neural development, such as cell proliferation, differentiation, and tissue regeneration. Disruption of this pathway following SAH may lead to abnormal glial cell activation and impaired coordination of cellular responses, thereby hindering the repair and recovery of damaged neural tissues. The enrichment of the oxidative phosphorylation pathway suggests that SAH may affect mitochondrial function, leading to energy metabolism disorders and exacerbating neural damage. Moreover, the MAPK signaling pathway plays a central role in neuroinflammation and cell survival regulation, and its activation has been closely associated with neuroinflammation following SAH (19). Earlier studies have highlighted PGRN's role in suppressing MAPK-associated inflammatory responses [18], indicating that it may modulate the activation of the MAPK signaling pathway in response to SAH, thereby affecting both inflammation and myelin integrity.

KEGG enrichment analysis revealed that genes differentially expressed in response to SAH were notably enriched in the MAPK and NF-κB signaling pathways. Both of these pathways are crucial in regulating neuroinflammation and cell survival, and they play significant roles in the neural damage caused by SAH (19). The NF-κB pathway, in particular, is a key mediator of inflammation-induced injury following SAH. Hemoglobin degradation products and oxidative stress resulting from SAH can activate NF-κB, leading to the release of pro-inflammatory cytokines (such as IL-6 and TNF-α) and chemokines (like MCP-1), which intensify neuroinflammation. Importantly, PGRN can inhibit TNF-α from binding to its receptor TNFR, preventing excessive NF-κB pathway activation, thus reducing the inflammatory response and promoting neuronal survival [19,20]. Additionally, the activation of NF-κB is closely related to microglial activation after SAH. Its excessive activation may lead microglia to produce neurotoxic effects. PGRN may influence NF-κB activity and thereby modulate inflammatory responses in microglia, thereby aiding myelin integrity. Therefore, we speculate that SAH may activate the MAPK and NF-κB signaling pathways, promoting inflammatory responses that may contribute to white matter injury, while PGRN may suppress the abnormal activation of the NF-κB signaling pathway, reducing neuroinflammation and promoting myelin integrity. This finding further supports the protective role of PGRN in SAH-related white matter damage and provides potential research directions for future targeted interventions involving PGRN.

Our study found that 14 days after SAH, significant lesions were present in the hippocampus and striatum of rats, suggesting that SAH can lead to long-term brain damage. Previous studies have shown that SAH can damage the brain's lymphatic system, leading to impaired cerebrospinal fluid circulation, brain edema, and a reduced ability to clear harmful substances, indicating that SAH may trigger a certain degree of irreversible damage [21]. Our results confirm this and suggest that PGRN may play a neuroprotective role in the process of white matter damage. The structural characteristics of myelin, including length, thickness, internodal length, and the composition of myelin proteins, are closely related to its function. Studies have shown that increased myelin thickness is often one of the markers of oligodendrocyte maturation, and this process is regulated by various factors, including axonal signals, the extracellular matrix, and microglial cells [22,23]. These factors regulate myelin formation, remodeling, and repair, ultimately affecting the transmission of neural signals and overall neural function [24,25].

In this study, rats with high PGRN expression exhibited significantly increased myelin thickness and a significant decrease in the g-ratio. This finding may reflect improved myelin structural integrity following PGRN overexpression. The results from transcriptomic analysis revealed that the differentially expressed genes after SAH were predominantly linked to processes like glial cell differentiation, myelin production, and immune system regulation. This provides further molecular support for the role of PGRN in promoting myelin integrity. Particularly, genes related to oligodendrocyte differentiation and myelin protein synthesis showed a significant upregulation after SAH, suggesting that PGRN may contribute to the preservation of myelin structure by regulating these key factors. Additionally, functional terms in GO enrichment analysis related to glial cell development and gliogenesis also indicate that myelin damage after SAH may be closely related to glial cell dysfunction. The upregulation of PGRN may improve white matter damage induced by SAH by supporting white matter structural integrity.

Cognitive dysfunction is one of the common neurological complications after SAH, which may be associated with white matter damage, inflammation, and impaired synaptic plasticity. These factors together contribute to the dysfunction of neural networks, further affecting cognitive functions such as learning, memory, and attention [26,27]. We observed a marked decline in cognitive abilities after SAH in this study, which was closely related to white matter damage and insufficient myelin integrity.

AL001 is a PGRN expression enhancer that effectively prevents PGRN degradation by blocking the interaction between sortilin and PGRN, thereby significantly extending its half-life and increasing PGRN levels in both brain tissue and serum by 2- to 3-fold [28]. In this study, AL001 exhibited significant neuroprotective effects in a SAH animal model. Specifically, AL001 treatment markedly upregulated PGRN expression, improved cognitive dysfunction, and promoted myelin integrity. The behavioral tests indicated that rats receiving AL001 treatment showed significant enhancements in learning and memory performance, reflected in quicker escape times, greater time spent in the target quadrant, and higher platform crossings. This suggests that elevating PGRN levels contributes positively to cognitive recovery. Transmission electron microscopy further revealed that AL001 promoted myelin structural restoration, suggesting its potential as a therapeutic agent for neurological impairments caused by brain injury. Particularly, AL001 holds promise in facilitating neural repair and cognitive recovery following acute brain injuries such as SAH and stroke.

Notably, AL001 has entered multiple phases of clinical research aimed at exploring its application in neurological diseases [28]. These studies further support its safety and potential efficacy, laying the groundwork for its future clinical translation in conditions such as SAH or stroke. Despite the promising results in preclinical models, further investigations are needed to assess its clinical applicability. In addition, future studies are needed to further investigate the molecular mechanisms between AL001, PGRN signaling, and downstream inflammatory pathways.

Of course, our study also has the following limitations: 1. Due to the scarcity of samples in clinical studies, the number of clinical samples included in this study is relatively small. In addition, the clinical cohort is relatively heterogeneous, which may limit the statistical power and generalizability of the findings. Therefore, the observations derived from clinical imaging and CSF analyses should be interpreted cautiously and will require validation in larger and more homogeneous patient cohorts in future studies. 2. This study primarily focused on the short-term effects after SAH and lacked long-term follow-up data. Neurodamage and cognitive dysfunction caused by SAH may change over time, so long-term follow-up data are crucial for assessing the sustained effects of PGRN treatment on neural recovery and cognitive function improvement. 3. In the KEGG pathway enrichment analysis of this study, although we considered the potential role of the NF-κB signaling pathway, the results showed that this pathway was not significantly enriched in the differential gene analysis. This may be related to the model, time points, or experimental design used in our study. For example, the NF-κB signaling pathway may play a role at different time windows after SAH, and thus, sampling at different time points or further validation in different animal models is needed to verify its involvement in the neurodamage and repair process. Furthermore, the involvement of the NF-κB signaling pathway may be more localized or act in conjunction with other pathways, which is why it was not significantly enriched at the overall transcriptomic level. 4. Although reduced phosphorylation of p65 was observed following PGRN treatment, gain- or loss-of-function approaches targeting NF-κB were not performed. Therefore, the present study demonstrates an association rather than definitive causal dependency of PGRN effects on NF-κB signaling. 5. The characterization of microglial activation relied on a limited set of markers (Arg-1, iNOS, and IL-1β), which does not fully represent the heterogeneity of microglial states following SAH. Future studies using broader marker panels or single-cell transcriptomic approaches will be necessary to more comprehensively define microglial phenotypes. 6. In addition, although MBP expression and ultrastructural analyses indicated improved myelin integrity after PGRN treatment, the present study did not directly assess oligodendrocyte lineage cells. Therefore, we cannot definitively distinguish between remyelination and preservation of existing myelin. Future studies incorporating specific markers of oligodendrocyte lineage cells will be necessary to more precisely evaluate remyelination processes.

## Conclusion

This study provides evidence suggesting a neuroprotective role of PGRN following subarachnoid hemorrhage (SAH), particularly highlighting its crucial function in promoting myelin integrity. Mechanistic exploration in this study suggests that PGRN treatment is associated with modulation of microglial inflammatory responses and reduced NF-κB activation following SAH. However, direct evidence demonstrating NF-κB dependency requires further investigation. Moreover, AL001, a small-molecule compound capable of upregulating PGRN expression, significantly improved myelin integrity, reduced brain lesion volume, and enhanced cognitive recovery in animal models. Notably, AL001 has already entered clinical trial stages, demonstrating favorable safety and promising therapeutic potential, thereby providing preliminary evidence supporting the potential of PGRN-targeted therapeutic strategies. These findings offer preliminary support for exploring interventions in white matter injury after SAH and suggest that PGRN or AL001 could be considered as potential targets for further study in SAH-related brain injury and cognitive dysfunction.

## Ethics approval and consent to participate

The human part of this study was approved by the Ethics Committee of the First Affiliated Hospital of Ningbo University (Approval No. 2024113A), and written informed consent was obtained from all participants. The animal experiments were approved by the Animal Ethics Committee of Ningbo University (Approval No. NBU20210333) and were conducted in accordance with institutional guidelines.

## Availability of data and materials

Data are contained within the article.

## Consent for publication

Not applicable.

## Author contributions

Conceptualization, X.G.,W.C. and C.Z.; methodology, Z.H., P.L., W.L., H.Y., Z.C., Y.Z., M.S., K.C.; writing—original draft preparation, Z.H.; writing—review and editing, C.Z. and X.G.; project administration, C.Z. All authors have read and agreed to the published version of the manuscript.

## Funding

This study was supported by grants from Ningbo Top Medical and Health Research Program (2022020304) and 10.13039/100007834Ningbo Natural Science Foundation (2022J215).

## Declaration of competing interest

The authors declare no conflict of interest.
